# Hydraulic Connectivity and Hydrochemistry Influence Microbial Community Structure in Agriculturally Affected Alluvial Aquifers in the Midwestern United States

**DOI:** 10.1021/acs.est.5c03155

**Published:** 2025-06-12

**Authors:** Hunter W. Schroer, Kendra Markland, Fangqiong Ling, Craig L. Just

**Affiliations:** † Civil, Architectural and Environmental Engineering, 14717 Missouri University of Science and Technology, Rolla, Missouri 65409, United States; ‡ 505147 United States Geological Survey, Iowa City, Iowa 52240, United States; § Department of Energy, Environmental, & Chemical Engineering, Washington University in St. Louis, St. Louis, Missouri 63130, United States; ∥ IIHR − Hydroscience and Engineering, 4083 University of Iowa, Iowa City, Iowa 52240, United States; ⊥ Department of Civil & Environmental Engineering, 4083 University of Iowa, Iowa City, Iowa 52240, United States

**Keywords:** groundwater, microbiomes, denitrification, N-DAMO, nitrification, tritium, biogeochemistry

## Abstract

Alluvial aquifers can provide ecosystem services and drinking water, but much remains unknown about human effects on aquifer microbiomes. Therefore, we used amplicon sequencing and hydrochemical characterization to pair microbial communities with environmental conditions across 37 alluvial aquifer wells. The study region spanned eastern Iowa and southern Minnesota (USA) and contained a combination of drinking water and monitoring wells. In terms of microbial ecology, dominant phyla across the wells included Proteobacteria, Bacteroidota, Patescibacteria, Planctomycetota, and Nitrospirota. Tritium, an indicator of infiltration and surface water influence, was the highest correlated variable with the Shannon index (α-diversity) by the Spearman rank sum (ρ = 0.60) and one of only four significant environmental variables in the constrained correspondence analysis. We built random forest regression models to predict tritium concentrations from microbial family relative abundance (held-out testing coefficient of determination (*R* ^2^) = 0.77 and mean absolute percentage error = 7%) and interpreted the models with Shapley additive explanation values. The most important families for predicting tritium concentrations were *Nitrosopumilaceae* and *Methylomirabilaceae*. Upwelling methane could contribute to the unusual coupling of ammonia oxidation by *Nitrosopumilaceae* with simultaneous nitrite-dependent methane oxidation by *Methylomirabilaceae*. Taken together, we illuminate the relationship among hydrochemistry, hydraulic connectivity, and alluvial aquifer microbiomes.

## Graphical abstract

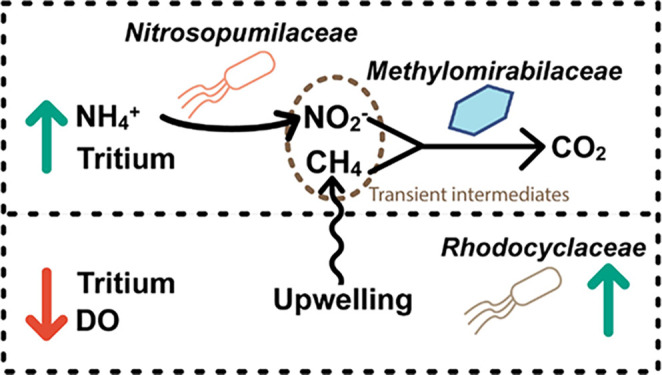

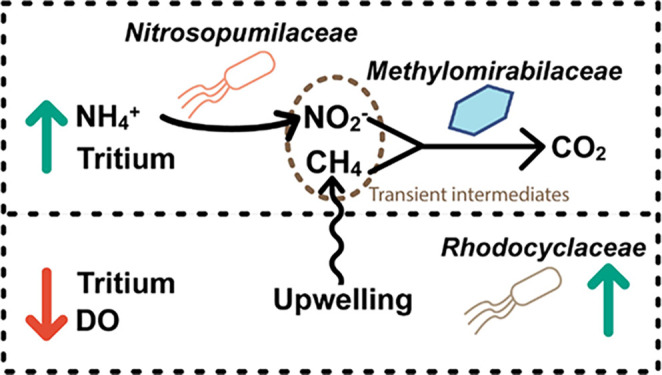

## Introduction

The deep subsurface hosts an estimated 15% of the total biomass on Earth, but its microbial ecology has received relatively little consideration.^1^ The huge microbial biomass in aquifers is responsible for the global cycling of critical elements like carbon, nitrogen, sulfur, and phosphorus, but an understanding of human effects on aquifer microbiomes and subsequent changes in nutrient cycling remains limited.^2–4^ Recent studies indicate that a combination of deterministic and stochastic forces shapes microbial community structure in groundwater aquifers, but the links remain tentative, with higher surface connectivity, transmissivity, and flow likely shifting the balance from deterministic to stochastic, immigration-driven microbiomes.^5,6^ Other studies have confirmed the importance of redox state and electron acceptor availability on microbiomes.^7,8^ On the other hand, in shallower aquifers, temporal and seasonal effects appear substantial, which could be due to environmental conditions or from the influence of constant inputs of soil microbes from infiltration.^9^ Furthermore, in a case study in the southern North China Plain, anthropogenic reactive nitrogen influenced the dominant microbial taxa and diversity along a groundwater flow path, indicating that the effect of exogenous pollutants was more important in shaping the ecological structure and function than groundwater circulation or rock–water interactions.^10^ Much remains unknown about microbial interactions in alluvial groundwater, especially in response to influences from surface water or infiltration.

High rates of surface infiltration can expose alluvial aquifers to anthropogenic contamination and a constant influx of microbes from overlying soil.^11–13^ In addition, rapid changes in surface infiltration volume and quality are likely to result in dynamic microbial communities that are less adapted to degrading pollutants, such as nitrate from plant fertilizers.^14^ Additionally, upwelling gases in alluvial aquifers could potentially expand redox interface zones where critical and transient chemical species interact and enable unique biochemical reactions such as methane oxidation and anaerobic ammonium oxidation (anammox). The influence of soil microbes could be higher in hydraulically connected zones, given the otherwise identical hydrochemistry, solely due to continuous soil microbe input due to precipitation and surface water influx.

Whereas other studies have begun to illuminate dominant microbial communities in alluvial aquifers, we sought to couple extensive hydrochemical data with microbial community surveys in agriculturally dominated alluvial aquifer groundwater wells, some of which provide private, domestic drinking water across three watersheds in eastern Iowa and southern Minnesota (Figure 1). Here, we clustered wells by hydrochemical properties and determined the influence of hydrochemistry on microbial community structure. We calculated correlations between measured chemical properties and observed microbial taxonomy, identifying factors that may influence nutrient cycling and microbiome function, including pathogen presence in these domestic and monitoring wells. Finally, we found that tritium in groundwater is a key indicator of water quality and related this surrogate for surface connectivity to taxonomy (α-diversity, potential pathogens, and families). Taken together, we demonstrate the importance of hydraulic connectivity for determining microbial community structure and potential function, show the effect of expanding critical redox zones on nutrient and pollutant cycling, and give new insights into the interplay among hydrochemistry, infiltration, and alluvial aquifer microbiomes.

**Figure 1:**
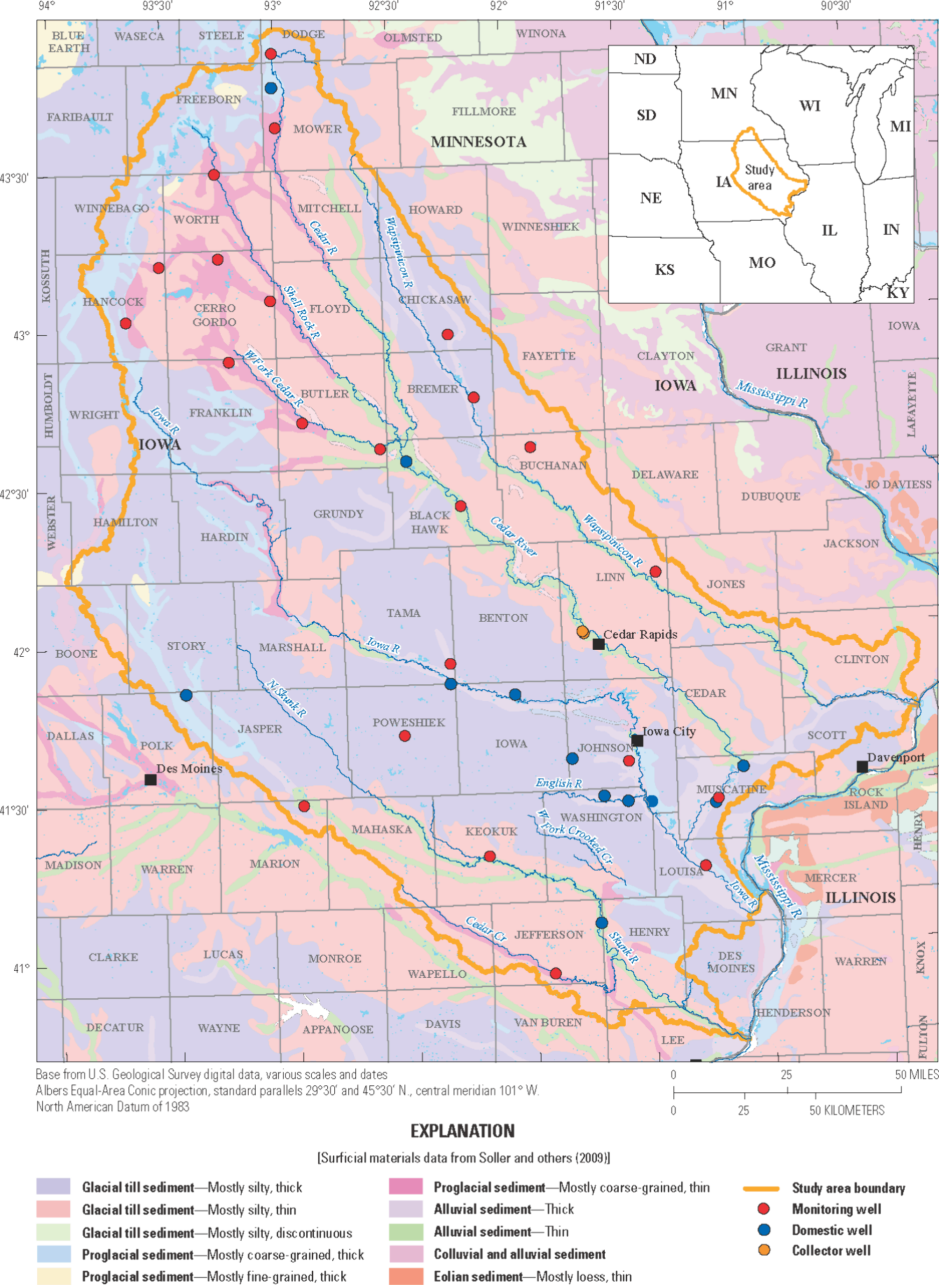
Monitoring, domestic, and collector wells sampled for this study and the U.S. Geological Survey’s Eastern Iowa Basins study unit, encompassing the Wapsipinicon, Cedar, Iowa, and Skunk River basins.^15^ Illustration courtesy of the U.S. Geological Survey.

## Materials and Methods

### Sample Collection and Processing

Alluvial aquifer groundwater samples were obtained from May to August 2017 from 37 wells in eastern Iowa and southern Minnesota (Figure 1) as part of the National Water-Quality Assessment (NAWQA) Program and were collected following the U.S. Geological Survey (USGS) National Field Manual for the Collection of Water-Quality Data.^16^ The NAWQA Program consists of domestic and municipal wells that could supply drinking water and monitoring wells screened in targeted aquifers strictly for the purpose of water-quality characterization. Water depth measurements were obtained using an electric tape before and after sampling. Wells were purged a minimum of three well-casing volumes until field measurements had stabilized before sample collection. On-site values for pH, dissolved oxygen (DO), specific conductance, water temperature, and turbidity were recorded by using a Eureka Manta+ water probe equipped with a flow-through cell (Eureka Water Probes, Austin, TX). Field values for DO below the limit of quantification (∼0.5 mg per liter [mg L^–1^]) were included in the analysis. A total of at least five values were recorded for each field measurement, with 5 min between recordings, until values were stable.

Domestic and municipal well samples were obtained via a direct connection to a spigot, and monitoring well samples were obtained via a submersible gear pump (Fultz Pumps, Inc., Lewistown, PA). Filtered samples for major ions, nutrients, and dissolved organic carbon (DOC) were collected at all sites and were filtered with a 0.45 μm pore-size capsule filter (Versapor High Capacity, Pall Corporation, Port Washington, NY) before preservation and overnight shipping to the USGS National Water Quality Laboratory in Denver, Colorado. Samples for tritium were analyzed at the University of Miami Tritium Laboratory in Miami, Florida. All water quality and water-level data are publicly available via the USGS National Water Information System (NWIS) Web Interface (10.5066/F7P55KJN).

Microbial samples, specifically for this research, were also collected at each well after all water-quality samples were collected. Autoclaved tubing (C-Flex, 0.635 cm inner diameter, Cole Parmer, Vernon Hills, IL) was attached to the sterilized spigot or the pump outlet. The sample was collected by filtering 1 L of groundwater through a 0.22 μm filter (Sterivex-GP Sterile Vented Filter Unit, Millipore Sigma, St. Louis, MO). Smaller volumes were filtered at sites with turbid water. A sterile, 10 mL syringe filled with air was used to remove excess water from the filter unit before capping with Luer Lock plugs. The capped filter cartridge was labeled and placed in a 50 mL conical centrifuge tube (Falcon, Thermo Fisher Scientific, Waltham, MA) for storage and transport. Each filter was then placed in a plastic bag and stored on ice before being returned to the laboratory. Filters were stored at −20 °C until further processing.

The microbial deoxyribonucleic acid (DNA) was extracted from the Sterivex filters after thawing on ice and was isolated using the DNeasy PowerWater Sterivex kit (Qiagen, Germantown, MD) according to the manufacturer’s instructions. Briefly, cells were lysed by vortexing, heating, and bead beating. Inhibitors were removed, and DNA was eluted from the filter prior to storage at −20 °C until further processing. The isolated DNA (1–50 ng per microliter [ng/μL], 20 μL) was shipped to Argonne National Laboratory, Environmental Sample Preparation and Sequencing Facility (Lemont, IL).

The v4 region of the prokaryotic 16S ribosomal ribonucleic acid (rRNA) gene was amplified (with 515F-806R primers)^17^ to prepare the amplicon libraries. Each polymerase chain reaction (PCR) mixture contained 9.5 μL of MO BIO PCR DNA-free water, 12.5 μL of QuantaBio’s AccuStart II PCR ToughMix (1x final concentration), 1 μL of the Golay barcode tagged forward primer (200 picomolar [pM] final concentration), 1 μL of reverse primer (200 pM final concentration), and 1 μL of template DNA. The PCR reaction was initiated at 94 °C for 3 min to denature the DNA, then 35 cycles at 94 °C for 45 s, then 50 °C for 60 s, and then 72 °C for 90 s, with a final extension time of 10 min at 72 °C. The amplicon libraries were quantified using PicoGreen (Invitrogen, Waltham, MA) and a plate reader (Infinite 200 PRO, Tecan, Switzerland). The amplicons were pooled into a single tube, with each amplicon represented in equimolar amounts. The pooled samples were cleaned using AMPure XP Beads (Beckman Coulter, Brea, CA) and quantified with a fluorometer (Qubit, Invitrogen, Waltham, MA). The pool was then diluted to 2 nM, denatured, and diluted to a final concentration of 6.75 pM with a 10% PhiX spike. Amplicons were sequenced on a 151 base pair [bp] × 12 bp × 151 bp Illumina MiSeq (Illumina, Inc., San Diego, CA) run.^17,18^

### Data Analysis

For hydrochemical analysis, we selected chemical properties from the USGS NWIS data for each well (Table 1). Nondetects were set to half the reported detection limit, and values that were not analyzed were set to the median value for that property across all wells. We performed agglomerative hierarchical clustering by scaling the data to zero mean and unit variance, calculating a Euclidean distance matrix, and clustering by Ward’s linkage, which has previously been effective for identifying natural groups in terminal restriction fragment length polymorphism data.^19^ We performed a principal component analysis (PCA) by scaling the data to zero mean and unit variance and using the PCA­() function in the scikit-learn module (v. 1.2.1) in Python (v. 3.10).

**Table 1:** Summary Statistics for Hydrochemical Data^a^

property	unit	mean	standard deviation	min	max	*n*	*n*, nondetects
specific conductance (EC)	μS cm^–1^	559	214	248	1,080	37	0
dissolved oxygen (DO)	mg L^–1^	3.9	3.4	0.1	10	37	0
pH	standard units	6.8	0.5	5.5	7.6	37	0
carbon dioxide	mg L^–1^	67	59	7	258	37	0
carbonate	mg L^–1^	0.2	0.2	0.05	1.3	37	9
bicarbonate	mg L^–1^	262	135	17.9	550	37	0
ammonia (NH_3_ + NH_4_^+^)	mg-N L^–1^	0.30	0.85	0.005	4.3	37	24
nitrate	mg-N L^–1^	7.2	8.5	0.02	33	37	11
orthophosphate	mg-PO_4_^3–^ L^–1^	0.28	0.52	0.024	3.2	37	0
dissolved organic carbon (DOC)	mg L^–1^	1.5	0.90	0.24	4.2	37	0
hardness	mg-CaCO_3_ L^–1^	270	106	91	548	37	0
noncarbonate hardness	mg-CaCO_3_ L^–1^	71	49	0	254	32	0
calcium	mg L^–1^	72	26	25	138	37	0
magnesium	mg L^–1^	22	11	6.6	56	37	0
sodium	mg L^–1^	12	20	1.2	100	37	0
sodium adsorption ratio	unitless	0.3	0.5	0.04	2.8	37	0
sodium fraction of cations	percent in equivalents of major cations	8	9	1	47	37	0
potassium	mg L^–1^	1.5	1.3	0.23	5.5	37	0
chloride	mg L^–1^	22	37	0.37	168	37	0
sulfate	mg L^–1^	22	16	0.05	60	37	0
fluoride	mg L^–1^	0.2	0.1	0.02	0.4	37	0
silica	mg L^–1^	21	6.8	5.9	36	37	0
iron, dissolved	mg L^–1^	685	1,761	5	9,450	37	14
manganese	mg L^–1^	68	122	0.2	556	37	10
tritium	pCi L^–1^	13	6.6	0.01	21	35	0
depth to water	m	4.2	5.3	0.39	25	30	0
turbidity	formazin nephelometric units	79	405	0	2,300	32	0
total dissolved solids	mg L^–1^	328	102	150	617	37	0
well depth	m	13	15	3.5	59	37	0

a Abbreviations: *n*, number; μS cm^–1^, microSiemens per centimeter; mg L^–1^, milligrams per liter; CaCO_3_, calcium carbonate; pCi L^–1^, picocuries per liter; m, meters.

We primarily used the DADA2 ^20^ (v. 1.16), phyloseq^21^ (v. 1.46.0), and vegan (v. 2.6.4) packages in R (v. 4.3.2) to process the raw 16S amplicon sequencing data. Paired-end FASTQ files were quality filtered (DADA2 maxEE = 2, where EE = sum­(10^(−*Q*/10)^) and *Q* is the quality score at each position) and left trimmed (0 and 18 bp for the forward and reverse reads, respectively), and the sequencing error rates were estimated from the data. Sequencing reads were then dereplicated, and the sequencing variants were inferred from the data. The forward and reverse reads were then merged using the overlapping region, an amplicon sequence variant (ASV) table was constructed and trimmed to include only sequences between 234 and 239 bp, and chimeras were removed. Finally, we used the ASV table to assign taxonomy based on the SILVA project database^22^ (v. 138.1), and we removed sequences classified as mitochondria and chloroplasts.

Next, we used a variety of rarefaction approaches to calculate diversity metrics and ordinations. To calculate the Shannon index (α-diversity), we first used the metagMisc R package (v. 0.5.0) to generate 1000 random phyloseq objects, each subsampled without replacement (rarefied) to the smallest sequencing depth (23,067 reads). We then calculated and used the mean α-diversity from the 1000 subsamples. To calculate the Bray–Curtis distance, we used the avgdist­() function in vegan to calculate the dissimilarity matrix from the rarefied ASV Table (1000 iterations, 23,067 reads) and the metaMDS­() function to create the nonmetric multidimensional scaling (NMDS) ordination of the microbial communities. For constrained correspondence analysis (CCA), we used the vegan ordistep­() function to build the CCA model from all of the available properties. This function begins by fitting the CCA of the microbial community with an intercept only and then sequentially adds (α = 0.05) or removes (α = 0.1) environmental variables based on permutation significance. We rarefied raw counts to an even depth of 23,067 reads and built the CCA model from the resulting subsampled ASV table, which was also used for all downstream analyses. We compared the NMDS and CCA microbial community ordinations to the hydrochemical PCA by Procrustes analysis using the vegan procrustes­() function. The rarefied ASV table was manipulated using phyloseq functions tax_glom­() to roll up ASVs and investigate higher taxonomic relationships and psmelt­() to use the agglomerated table for plotting and exporting for predictive modeling.

We used adaptive lasso^23,24^ and random forest regression models implemented with the scikit-learn library^25^ in Python to predict tritium concentrations from family-level taxonomic data. First, we filtered to keep only families that were detected in more than 80% of the samples (29 phyla) and randomly split the data with 80% for training (*n* = 29) and 20% (*n* = 8) for hold-out testing. Using adaptive lasso with ridge regression for initial parameter estimation^24^ resulted in a model with no significant multiple linear regression coefficients (data not shown). Therefore, we fit the family data with a random forest regression model to predict tritium concentrations. We used a leave-one-out cross-validation (the ideal strategy for small sample sizes)^26^ grid search for hyperparameter tuning with the mean absolute percentage error (MAPE) as the optimization parameter. All grid search parameters are given in Table S1. We then trained a random forest regression model on the training data and used this model to predict tritium concentrations in the held-out testing data.

We evaluated the random forest regression model on the testing data with the coefficient of determination (*R* ^2^) and the MAPE, which are defined as

R2=1−∑i=1n(yi−y^i)2∑i=1n(yi−y̅)2 1

where *n* is the number of observations, *y*_*i*_ is the actual value for sample *i*, *ŷ*_*i*_ is the predicted value, and *y̅* is the mean value.

MAPE=1n∑i=1n|yi−y^iyi| 2

To uncover individual family contributions to the predicted tritium concentration, we used Shapley additive explanation (SHAP) analysis (*shap* Python package). SHAP values are the contributions of each family for each individual prediction (i.e., local explanation) but can also be generalized to the entire data set (i.e., global explanation).^27^ Higher absolute SHAP values over the entire model translate to higher model importance. Due to its ease of calculation and interpretation, SHAP values have been commonly used in other environmental studies.^23,28–31^ We first trained the SHAP kernel explainer on the training set and then fit the explainer to the test set. We then used the *shap* package to produce summary and waterfall plots for the testing data set. For neutral community modeling, we used the *minpack.lm* library to fit the taxonomic family occurrence frequency versus mean family relative abundance to a beta distribution using a bounded Broyden–Fletcher–Goldfarb–Shanno (L-BFGS-B) maximum likelihood estimator (*stats4* library) and calculated a 95% confidence interval on the distribution.^32,33^

Visualizations were produced using a combination of ggplot2 (v. 3.5.0), matplotlib (v. 3.7.0), and GraphPad Prism (v. 10.0.2), and figures were compiled in Inkscape (v. 1.3.2) or Adobe Illustrator (v. 28.6). All of the code is available at github.com/hunter-schroer/IA-groundwater-microbiome. Amplicon sequencing data were deposited as Bioproject PRJNA1225088 at the National Center for Biotechnology Information (USA).

## Results and Discussion

### Hydrochemistry

The variations in well type and depth resulted in a range of values across the measured hydrochemical properties (Table 1 and Figure 2). For example, well depth ranged from 3.5 to 59 m (m), DO ranged from 0.1 to 10 mg L^–1^, pH ranged from 5.5 to 7.6 standard units, and nitrate concentrations ranged from below the detection limit of 0.04–33 mg L^–1^ (Table 1). In general, the concentrations of electron acceptors and redox indicators followed expected patterns with depth (plotted in Figure 2A). At shallower well depths, DO and nitrate concentrations were the highest and most variable. Generally, DO and nitrate concentrations decreased quickly with depth and were minimal below 25 m. DO and nitrate are well-known electron acceptors and readily reduced by heterotrophic respiration of organic matter.^34^ In the oxic zone (well depth less than 25 m), DO concentrations had a mean of 4.5 and standard deviation of 3.4 mg L^–1^, nitrate was 8.6 ± 8.6 mg L^–1^, dissolved manganese was 62 ± 128 μg per liter (μg L^–1^), dissolved iron was 167 ± 488 μg L^–1^, and sulfate was 23 ± 14 mg L^–1^. In contrast, in the iron- and sulfate-reducing zone below 25 m, DO concentrations had a mean and standard deviation of 0.6 and 0.9 mg L^–1^, respectively, nitrate was 0.0 ± 0.0 mg L^–1^, dissolved manganese was 101 ± 84 μg L^–1^, dissolved iron was 3360 ± 3261 μg L^–1^, and sulfate was 13 ± 24 mg L^–1^. Manganese generally decreased with depth. As DO and nitrate concentrations declined, dissolved iron also increased, indicating iron-oxide reduction to soluble Fe^2+^.^35^ In the presence of DO and nitrate, iron reduction is less thermodynamically favorable, and Fe^2+^ is rapidly oxidized and precipitated as insoluble iron oxides in the presence of oxygen.^36,37^ As expected, as the dissolved manganese concentrations decreased, soluble iron increased. Like those of DO and nitrate, sulfate concentrations generally decreased with depth. In general, the pH and alkalinity increased with depth, also consistent with the observed redox gradient. Redox conditions closely matched our expectations. Each deeper well contained measurable sulfate or DO, likely limiting methanogenesis and explaining a lack of detectable methane.^38^

**Figure 2:**
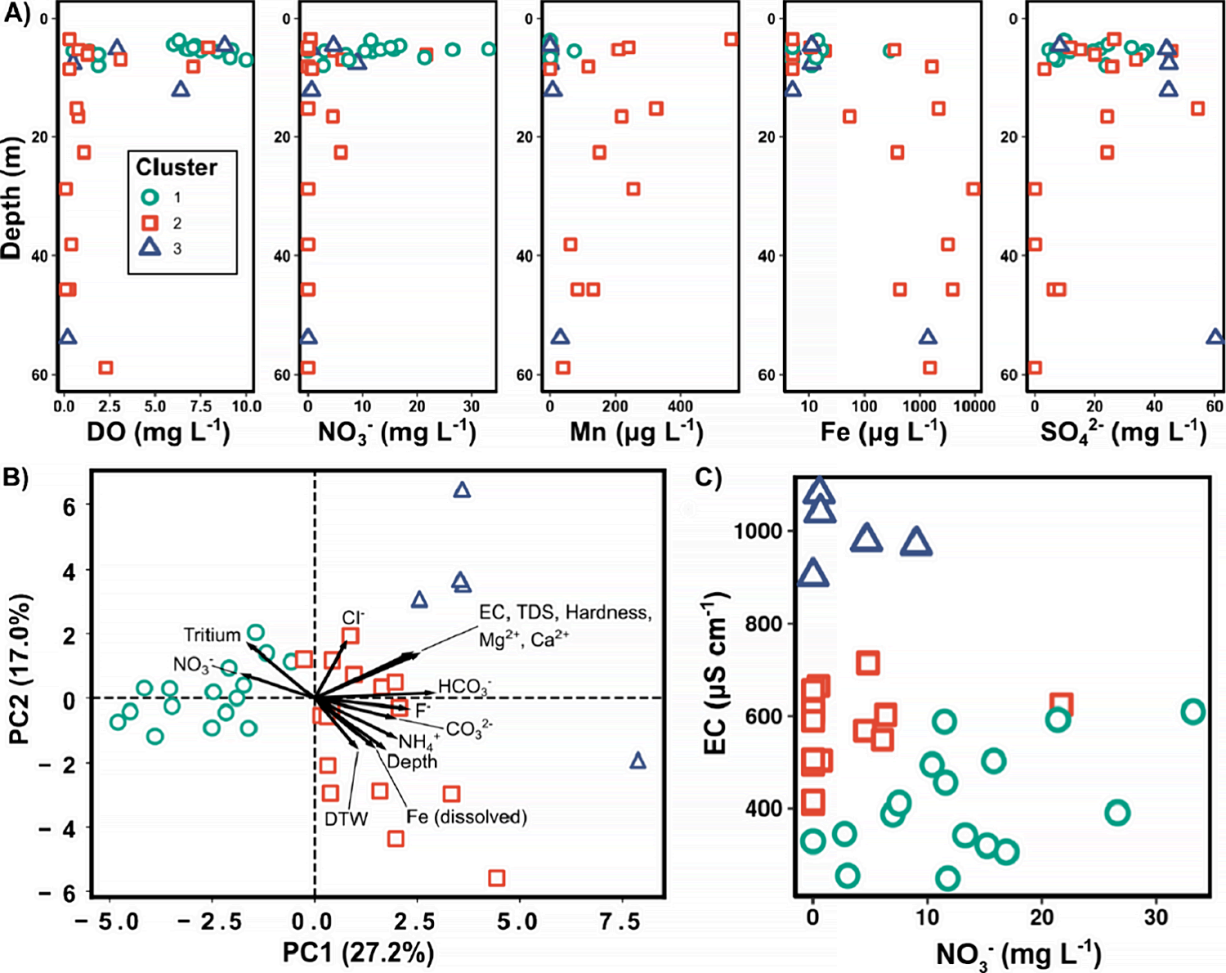
Comparing well hydrochemistry by cluster analysis and plotting select properties by depth, PCA, and the two key properties that differentiate well chemistry by cluster. Clusters were determined from agglomerative hierarchical feature clustering (Ward’s linkage) of the normalized hydrochemical data set and are indicated by shape and color of the symbols, with each symbol representing a single well sample. (A) Depth profiles for concentrations of redox indicators. (B) PCA biplot of the first two principal components of the hydrochemical data (44.2% of the total variance). Arrows are loadings corresponding to the largest 15 hydrochemical property eigenvectors. (C) Measured values of specific conductance (EC) vs nitrate with clusters indicated. Ca^2+^, calcium; CO_3_^2–^, carbonate; DO, dissolved oxygen; DTW, depth to water; EC, specific conductance; Fe, iron; HCO_3_^–^, bicarbonate; Mg^2+^, magnesium; Mn, manganese; NH_4_^+^, ammonium; NO_3_^–^, nitrate; SO_4_^–^, sulfate; TDS, total dissolved solids; L, liter; mg, milligram; μg, microgram; μS, microSiemens; cm, centimeter; m, meter.

Using agglomerative hierarchical clustering by Ward’s method, we grouped wells by hydrochemical properties, which yielded three sensible clusters. The first two clusters were predominantly separated by redox indicators (Fe and NO_3_^–^) and depth. The third cluster was primarily separated from the other two by EC, and properties were correlated to EC (Figure 2B). Indeed, examining the raw EC data as a function of nitrate alone almost completely separated the clusters, and cluster 3 was differentiated by high EC values (Figure 2C). Cluster 1 had the highest nitrate values coupled with lower EC at lower nitrate values.

We next examined the hydrochemical data by PCA after centering and scaling each property by standard deviation. The first two principal components fully separated the clusters determined by hierarchical feature clustering and can explain 44.2% of the total variance in the data (Figure 2B). In a similar previous analysis, the first two principal components could explain 41% of the hydrochemical variation across three groundwater basins in the Death Valley Regional Flow System.^6^ Two other studies utilizing PCA to explain hydrochemical variance had a higher explanatory power with the first two principal components but took advantage of additional chemical characteristics, such as fluorescence and stable isotopes that were not collected here.^39,40^ Important hydrochemical variables in the PCA include EC (and correlated variables TDS, hardness, Mg^2+^, and Ca^2+^), surface connectivity (depth to water [DTW], well depth, and tritium), alkalinity, anions potentially associated with rock weathering (Cl^–^ and F^–^),^41,42^ nitrogen species (NH_4_^+^ and NO_3_^–^), and dissolved iron.

In our study area, where agriculture dominates land use, nitrate is expected to be derived from surface water runoff. Tritium is a conservative tracer of groundwater age and can therefore measure the extent of infiltration of precipitation or surface water compared to premodern groundwater, and it derives primarily from atmospheric nuclear weapons testing from 1953 to 1963.^43^ Therefore, cluster 1 is indicative of a high percentage of infiltration and surface water influence compared to those of the other clusters based on the hydrochemical data alone.

### Microbial Ecology

The microbial communities present in the alluvial groundwater were diverse across samples and were generally consistent with those of previous studies. Surprisingly, there were few core taxa across all of the samples. For example, only two ASVs were present in more than 70% of the samples, only five ASVs were present in more than 60% of the samples, and none were common to all samples (Figure S1). For comparison, while examining a slightly lower taxonomic resolution, a recent study found that 26 operational taxonomic units (OTUs) were common across 10 groundwater wells, comprising 21% of the total microbial community on average.^5^ An additional study found that 31% of the bacterial sequences were shared across suspended growth samples.^7^ However, our study spanned over 300 km and three watersheds, compared to less than 100 km and a single aquifer in each of these previous studies. Taken together, geographical distance and separate watersheds contribute to the higher diversity of microbial communities.

At the phylum level, filtering by common occurrence (arbitrarily selected as presence across 80% of the samples) yielded 29 phyla (Figure S2), which accounted for most of the microbial community across the samples. Similar to other alluvial aquifers, dominant phyla included Proteobacteria, Bacteroidota, Patescibacteria, Planctomycetota, and Nitrospirota.^5,44–46^ Unexpectedly, we noted common observation of Methylomirabilota and Verrucomicrobiota, which are not common among other groundwater aquifers.^5,7,10,38^ Looking at the top five common phyla by median abundance, we observed Bacteroidota, Nanoarchaeota, Patescibacteria, Proteobacteria, and Verrucomicrobiota (Figure S2). At the family level of taxonomy, filtering by common occurrence (arbitrarily selected as presence across 90% of the samples) yielded 27 families, making up about 20–35% of the microbial community in each sample (Figure S2). The five common families with the highest median relative abundance were *Nitrosomonadaceae*, *Nitrospiraceae*, *Omnitrophaceae*, and *Pedosphaeraceae*, and a family was classified as *GW2011_GWC1_47_15*, which falls within the Nanoarchaeota phylum. In terms of the most abundant families (not filtering for common detection), the five families with the highest relative abundance in any one sample were *Gallionellaceae*, *Mycobacteriaceae*, *Methylophilaceae*, *Omnitrophaceae*, and *Crocinitomicaceae.*

The predominant taxa matched previous groundwater microbiomes, with a few notable exceptions. At the phylum level, our observation of abundant Bacteroidota matches previous results implicating this phylum as important in oligotrophic environments, using diverse carbohydrate transport and degradation systems to cycle complex carbohydrates.^44^ Patescibacteria and Nanoarchaeota are known for small cell sizes and may be preferentially enriched in our suspended samples compared to sediment-localized microbes.^47,48^ Verrucomicrobiota have been gaining attention as predominant soil and marine microbes,^49,50^ but the ecological role of this phylum in groundwater and its importance in this study remain indeterminant. Perhaps an influx of soil-derived microbes contributes to the prevalence of the Verrucomicrobiota observed here. At the family level, *Omnitrophaceae* and *Pedosphaeraceae* (both within the phylum Verrucomicrobiota) were commonly detected with high relative abundance. *Omnitrophaceae* appear to be predatorial or parasitic, hyperactive, and of unusually small cell size.^51^ *Pedosphaeraceae* appear to be primarily soil- and plant root-zone-associated and could therefore result from infiltration.^52,53^ On the other hand, well-known nitrogen-cycling bacteria (*Nitrospiraceae* and *Nitrosomonadaceae*) were unsurprisingly abundant across the groundwater samples. *Nitrospiraceae* is the most common nitrite-oxidizing family,^54^ and *Nitrosomonadaceae* is made up of ammonia-oxidizers.^55^ Ammonia-oxidizing archaea *Nitrosopumilaceae* were present in relative abundances from nondetect up to 5% of the total microbial community. *Nitrosomonadaceae* and *Nitrosopumilaceae* were likely responsible for ammonia oxidation in the relatively shallow alluvial aquifers of this study, which are surrounded by heavily agricultural lands and, therefore, ammonia fertilizer inputs.

### Hydrochemistry Influences Microbial Community Structure

We next related the hydrochemical properties and clusters to microbial community composition. An NMDS plot of the Bray–Curtis distance (β-diversity) did partially demonstrate that hydrochemical properties influenced the microbiome composition (Figure 3A). Specifically, clusters 1 and 2 (assigned only by well hydrochemistry) were almost entirely separated using microbiome data. However, cluster 3 was intermingled with the other clusters, indicating that the measured hydrochemical properties could not fully explain the microbiome composition. Cluster 3 was separated by EC and correlated properties in the hydrochemistry (Figure 2B,C), which are likely less deterministic for microbiome assembly than the measures of redox chemistry and infiltration that separated clusters 1 and 2.

**Figure 3:**
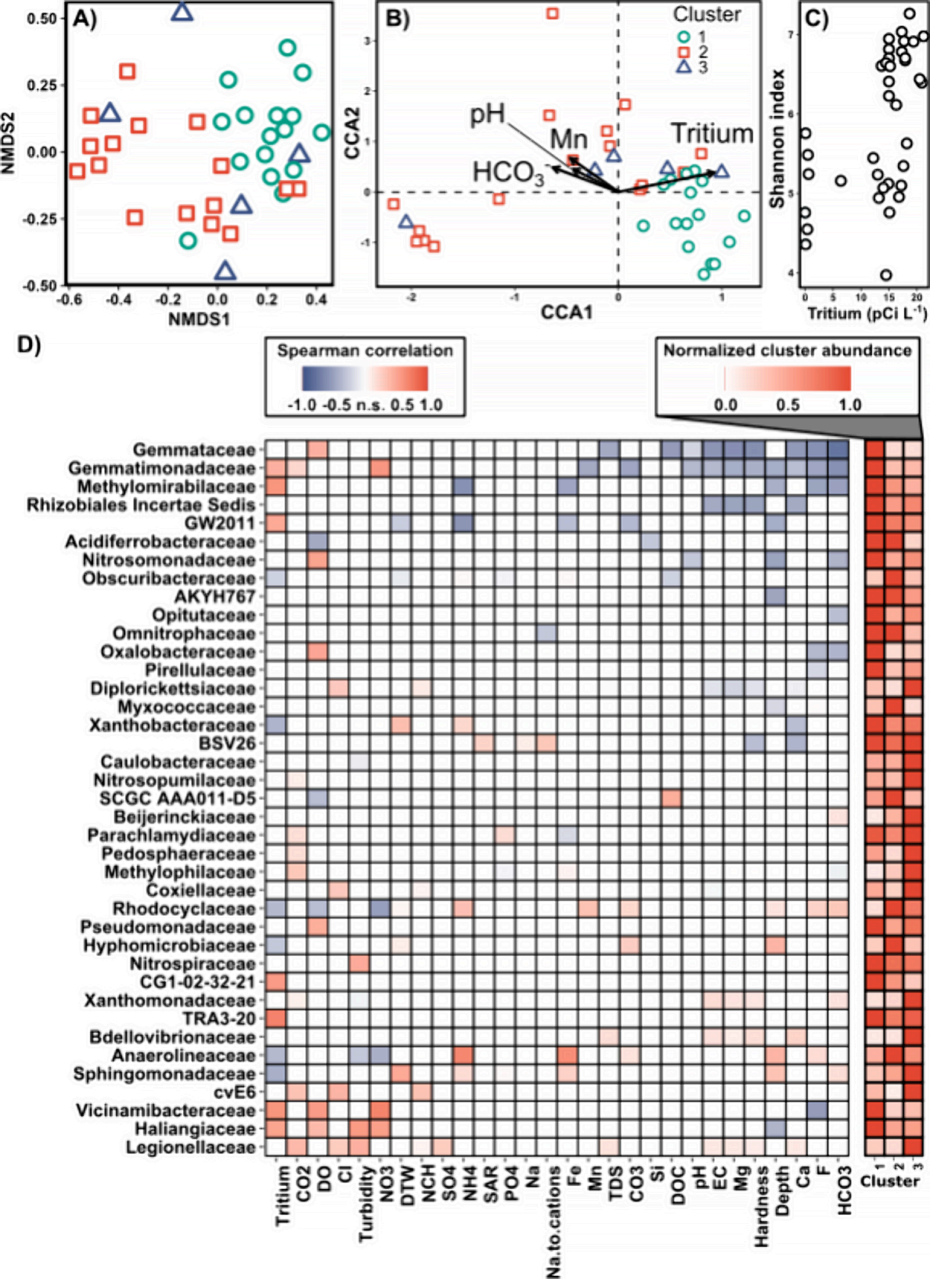
(A) NMDS ordination of the Bray–Curtis distance among microbiomes. Symbols and colors represent clusters from agglomerative hierarchical clustering of hydrochemical data, with each symbol representing a single well sample. (B) CCA ordination of microbiomes fit to hydrochemical properties determined to be significant (*p* = <0.05) from stepwise permutation analysis. Constrained inertia: 3.1; total inertia: 23.3 (13% constrained). (C) Shannon index of each sample versus well tritium concentration (Spearman's rank sum correlation: ρ = 0.60, *p* = <0.001). (D) (left) Spearman's correlation heatmap of taxonomic families present in more than 80% of the samples and hydrochemical properties that have at least one significant correlation and (right) mean relative abundance of each family normalized to the highest mean abundance of that family in any cluster. Ca, calcium; Cl, chloride; CO2, carbon dioxide; CO3, carbonate; Depth, well depth; DO, dissolved oxygen; DOC, dissolved organic carbon; DTW, depth to water; EC, specific conductance; F, fluoride; Fe, iron; HCO3, bicarbonate; Mg, magnesium; Mn, manganese; Na, sodium; Na.to.cations, sodium ratio of total cations; NCH, noncarbonate hardness; NH4, ammonia (ammonia + ammonium); NO3, nitrate; n.s., not significant (*p* = 0.05); pCi L^–1^, picocuries per liter; PO4, orthophosphate; SAR, sodium adsorption ratio; Si, silica; SO4, sulfate; TDS, total dissolved solids.

Next, we built CCA models to sequentially identify significant hydrochemical properties to constrain microbiome ordination. Four chemical properties were significant (α = 0.05) by permutation, including tritium, bicarbonate, pH, and manganese (Figure 3B). Surprisingly, the four properties explained only 13.2% of the CCA variance, leaving the vast majority of the variance unconstrained. Cluster 1 was differentiated in the plot, but clusters 2 and 3 were intermingled. Since EC and the associated properties (hardness, TDS, etc.) that were the highest in cluster 3 wells were apparently not deterministic for microbiome assembly, cluster 3 was not differentiated by CCA, unlike in the hydrochemistry PCA plot, where cluster 3 separated from cluster 2. Finally, we compared the NMDS (unconstrained) and CCA ordinations to the hydrochemical PCA with Procrustes analysis, and the ordinations were not significantly different (*p* < 0.001, NMDS; *p* < 0.001, CCA), further indicating that hydrochemistry at least partially explained the microbiome assembly.

Looking deeper into the significant hydrochemical properties yielded insight into the microbial community assembly. For example, the infiltration of precipitation, as indicated by tritium, is expected to affect nutrient concentrations and microbial population dynamics. Despite its use as a conservative tracer for groundwater age, tritium has been sparsely used as an indicator of microbial community analysis. We found one study that suggested tritium was important for predicting microbial phospholipid fatty acid profiles,^56^ but there are no apparent studies relating tritium as a water-quality property to the taxonomic composition of microbiomes. In addition to constraining the community ordination, tritium had the highest Spearman correlation to the observed α-diversity (Shannon index, Figure 3C), suggesting that infiltration of new water resulted in the greatest microbiome diversity. Our results indicated that infiltration of precipitation and surface water is an important driver for microbial community structure. Immigration of new microbes can result in higher microbiome diversity, such as that observed in a recharge area versus downstream flow.^5^ In addition, higher groundwater flux limits deterministic assembly, presumably because nutrient conditions are more variable and immigrant microbes are continuously input to the system.^6^ Local heterogeneity in our study region could have led to higher localized groundwater flux and transmissivity, minimizing deterministic community assembly and favoring microbial migration as a driver of community structure.^6,57^

In addition to tritium, bicarbonate, pH, and manganese were the remaining significant variables in the CCA. Bicarbonate is also a key substrate for autotrophs, potentially differentiating niches for auto- or heterotrophic growth or controlling pH buffering.^58,59^ Further, bicarbonate has similarly been shown to correlate with microbiomes by redundancy analysis across three Chinese aquifers.^60^ Gradients of pH are also critical drivers for microbial ecosystem dynamics.^61–63^ Again, separate studies found pH to be a significant environmental variable for microbial CCA and redundancy analysis across diverse aquifers.^38,60^ Finally, manganese is an important element in redox cycling,^64^ and, like nitrate, can explain almost all of the hydrochemical clustering in combination with EC (Figure S3).

We wondered whether taxonomic families were associated with individual hydrochemical properties, giving clues about niche partitioning and drivers behind community assembly under different environmental properties. Filtering families by presence in 80% of the samples, we calculated the Spearman correlation of the common families with the hydrochemical properties (Figure 3D). Tritium (13 correlated families), well depth (11), DO (10), and bicarbonate (10) were the hydrochemical properties with the highest number of correlated families. We were surprised that redox indicators like nitrate (5 correlated families), iron (5), manganese (2), and sulfate (1) did not have many correlated families, given the importance of redox and the associated growth and energy strategies in niche differentiation. *Gemmataceae* and *Gemmatimonadaceae* were the most prevalent in cluster 1 and were among the families with the greatest correlations to various hydrochemical properties, potentially indicating more niche differentiation or specific metabolic and ecosystem requirements. Both were negatively correlated with properties associated with rock weathering (e.g., calcium and fluoride) and positively associated with shallower, surface water-influenced wells (e.g., tritium, DO, and nitrate). *Vicinamibacteraceae* and *Haliangiaceae* followed the same patterns, having the highest prevalence in cluster 1 and positive correlations with tritium, DO, and nitrate and negative correlations with fluoride or depth. *Vicinamibacteraceae* and *Haliangiaceae* are thus far predominantly associated with soil,^65–67^ further suggesting the import of tritium as an indicator of infiltration, as these taxa are likely immigrants from soil environments. On the other hand, *Rhodocyclaceae* and *Anaerolineaceae* followed the opposite patterns, closely associating with cluster 2, decreased tritium and nitrate, and increased fluoride and well depth, indicating that these taxa were more associated with rock weathering and older, deeper aquifer conditions. Families most associated with cluster 3 included *Nitrosopumilaceae*, *Pedosphaeraceae*, *Methylophilaceae*, *Bdellovibrionaceae*, *Sphingomonadaceae*, and *Legionellaceae.*

### Random Forest Models Predict Tritium Concentrations from Taxonomic Families

Based on the correlation between tritium and α-diversity, the correlation between tritium and family abundances, and the significance of tritium in constraining the community ordination by CCA, we sought to further investigate the tie between tritium and the microbial community structure. We fit abundant families (detected in 80% of the samples, *n* = 27) to predictive models for tritium concentration. We fit a random forest regression model to the training data (80% of the samples) and used leave-one-out cross-validation for hyperparameter tuning. The resulting model had an *R* ^2^ of 0.77 and a MAPE of 6.8% on the held-out testing data (Figure 4A). The model results were promising and surprising given the small training data set (*n* = 29), including promising generalizability (training *R* ^2^ of 0.85 and MAPE of 20.2%).

**Figure 4:**
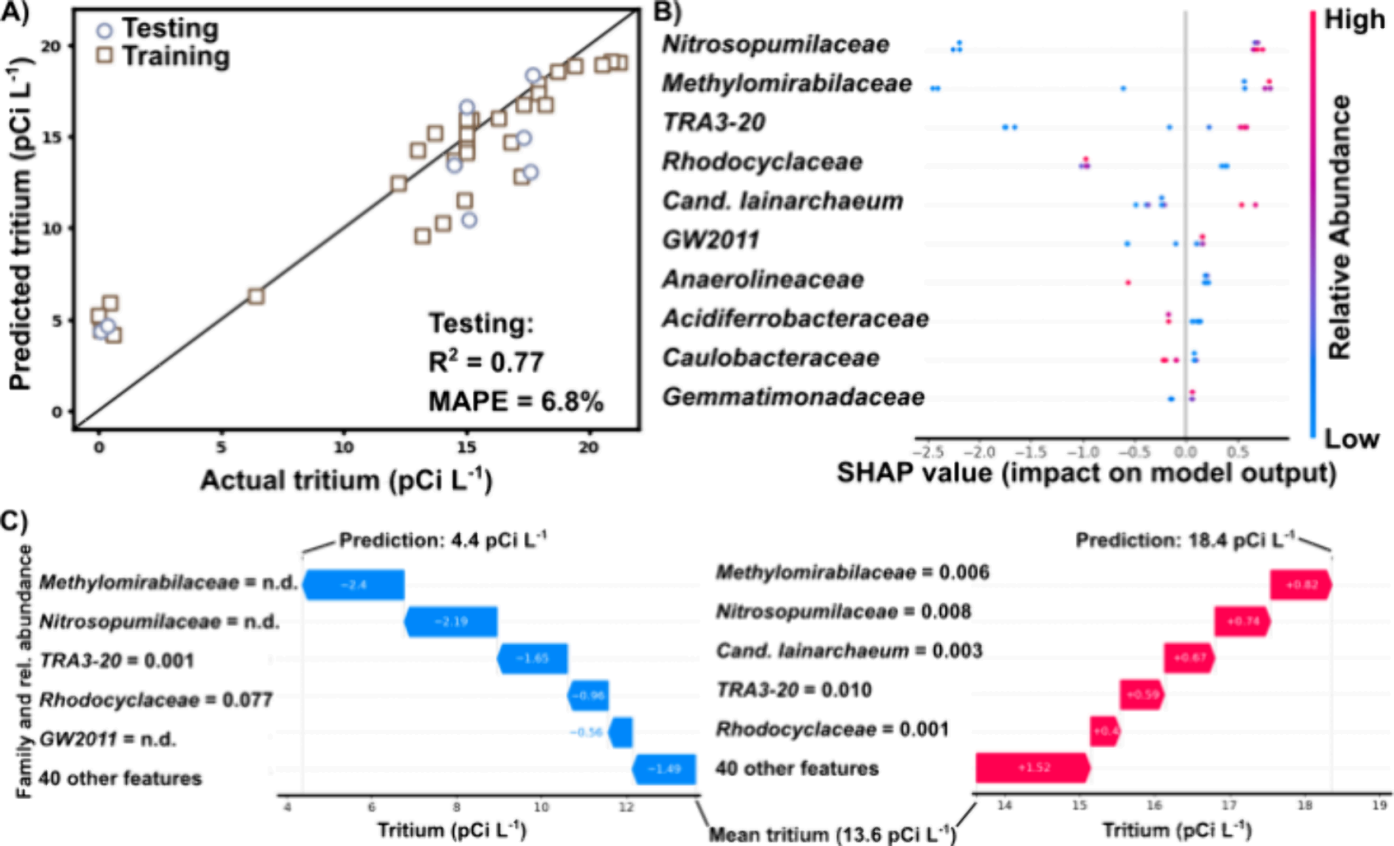
(A) Random forest regression of tritium predicted by family-level microbial communities with hold-out testing prediction metrics, where each point is a single well sample. (B) Shapley (SHAP) values as a function of relative abundance for each taxonomic family. (C) SHAP waterfall plots of two representative testing data points, where the relative abundance of each family contributes additively to the predicted tritium value for each sample in the random forest model. MAPE, mean absolute percent error; *R* ^2^, coefficient of determination; pCi L^–1^, picocuries per liter; SHAP, Shapley value.

We used SHAP values to interpret the random forest model. SHAP values are an interpretable approximation of the original model that derive from game theory.^68^ Each prediction begins from the mean, or expected, value of the predicted variable, and then each feature (here, family) contributes additively to the predicted value.^68^ Overall, SHAP values can be interpreted for feature importance, taking the entire testing set into account (Figure 4B), or for local importance, investigating the additive contributions of each family for individual samples (Figure 4C). In the summary (Figure 4B) and waterfall plots (Figure 4C), the most important families are listed at the top, with decreasing importance moving down the plots. Two representative model predictions are shown in Figure 4C, with the first prediction being 4.4 pCi L^–1^ and the second prediction being 18.4 pCi L^–1^, to demonstrate the effect of each family on representative low and high predicted tritium concentrations.

Notably, SHAP values indicated that the families most important for tritium prediction were *Nitrosopumilaceae*, *Methylomirabilaceae*, and *TRA3–20* and *Rhodocyclaceae*, both of which belong to the order Burkholderiales. At high relative abundances of *Nitrosopumilaceae*, *Methylomirabilaceae,* and *TRA3–20,* the model predicted more tritium, and at higher values of *Rhodocyclaceae*, the model predicted lower tritium concentrations (Figure 4B,C). In terms of *Nitrosopumilaceae*, two genera were present (*Nitrosarchaeum* and *Candidatus Nitrosotenuis*), with *Nitrosarchaeum* dominating with the presence in 29 of the 37 samples at relative abundances up to 4%. Presumably, the *Nitrosopumilaceae* are soil-derived ammonia-oxidizing archaea.^69–71^ The family *Methylomirabilaceae* was dominated by the genus Candidatus Methylomirabilis, which was present in 26 of the 37 samples, with relative abundances from nondetect to 1.2%. *Methylomirabilaceae* is an unusual and recent addition to the known nitrogen and oxygen-cycling organisms, responsible for producing dioxygen from nitric oxide, and using the oxygen to oxidize methane in otherwise anaerobic environments.^72^ The most commonly studied *Methylomirabilaceae* require nitrite for intra-aerobic methane oxidation.^72,73^ One explanation for the co-occurrence of *Methylomirabilaceae* and *Nitrosopumilaceae* in tritium-laden samples could be that high surface connectivity widens the potential redox zone where *Nitrosopumilaceae* have access to ammonia and oxygen or are present as soil immigrants, and *Methylomirabilaceae* can access the resultant nitrite, as well as upwelling methane (Figure 5). Alternatively, local microheterogeneity could lead to localized methanogenic zones such as a pocket of organic material. Since the metabolic intermediates nitrite and methane are transient and difficult to detect, here, we must rely solely on the taxonomic composition to infer microbial activity without further functional data, like transcriptomics or proteomics.

**Figure 5:**
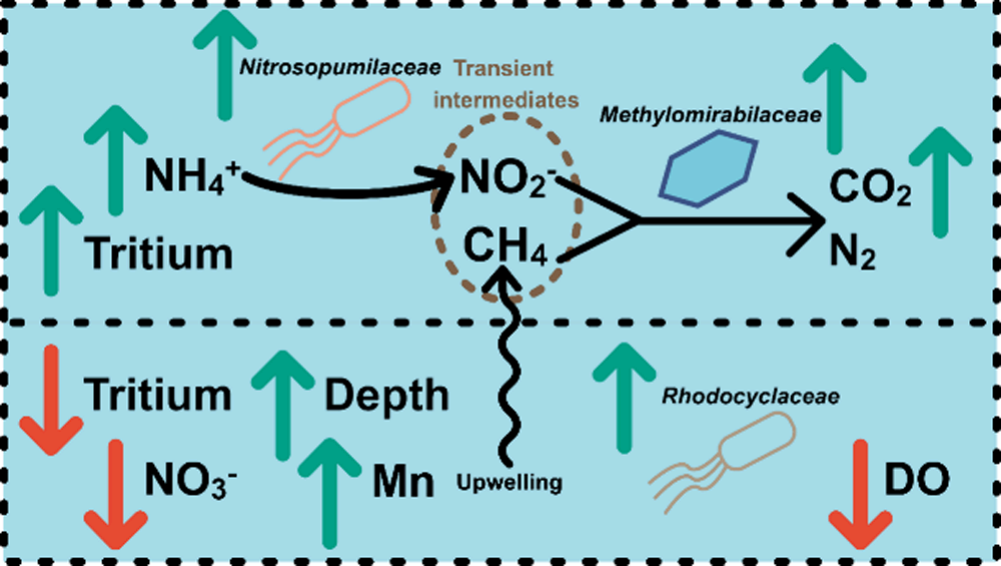
Conceptual model of higher tritium zones (top), where upwelling methane allows for coexistence of nitrite and methane, and lower tritium zones (bottom), where lower influence from surface water and infiltration leads to manganese-reducing conditions. Green arrows indicate either higher tritium, a positive Spearman rank sum correlation between the relative abundance of *Nitrosopumilaceae*, *Methylomirabilaceae*, or *Rhodocyclaceae* and the associated hydrochemical property, or the phylum’s influence on tritium prediction from the random forest model. Red arrows indicate either lower tritium or negative Spearman's rank sum correlations between the relative abundance of *Rhodocyclaceae* and nitrate and DO.

We fit a neutral community model to the mean family abundance and frequency data across the samples to determine which families fit the assumption of neutral community assembly by stochastic reproduction, death, and immigration (Figure S4).^32,33^ Overall, the family occurrence frequency was well described by the neutral community model (*R* ^2^ = 0.69). Notably, *Nitrosopumilaceae* and *Methylomirabilaceae* were inhibited in certain local communities (less prevalent than expected from neutral models, α = 0.05). The lower-than-expected occurrence of *Nitrosopumilaceae* and *Methylomirabilaceae* suggests that environmental selection limited the prevalence of these two families to a greater extent than families lying within the confidence interval for the neutral community model.^32^ Given their importance in predicting tritium concentrations and the potentially unique metabolic niche in which *Nitrosopumilaceae* and *Methylomirabilaceae* could feasibly coexist, it logically follows that their prevalence is tied to hydrochemistry to a greater extent than most families (i.e., the assembly of these two families is primarily deterministic rather than immigration-driven).

For phyla inversely related to tritium, *Rhodocyclaceae* (primarily made up of genera *Candidatus Accumulibacter*, *Dechloromonas*, *Denitratisoma*, *Ferribacterium*, and *Sulfuritalea*) is primarily composed of facultative or strict anaerobes that accumulate polyphosphate and reduce perchlorate, nitrate, and iron,^74–77^ giving weight to tritium as an indicator of oxygen infiltration and as a conservative tracer of oxidized waters.

### Potential Pathogens in Alluvial Aquifer Wells

Finally, we wanted to investigate the occurrence of potential pathogens in alluvial groundwater samples and the relations between potential pathogens and hydrochemical properties, given that some wells serve as domestic drinking water sources. Given the limited ability of short-read 16S rRNA sequencing to determine strain or even species-level taxonomic resolution, we note that metagenomic sequencing or strain-specific primers for targeted pathogen amplification are needed to accurately quantify the potential ingestion risk. Given these limitations, detection of *Mycobacteriaceae* (especially at a high relative abundance of 30% in a single sample) and *Legionellaceae* warrants further investigation into drivers of their occurrence and potential risk of exposure beyond this single sampling event in a relatively small number of wells (Figure S5). The *Legionellaceae* family was common among samples (95% detection frequency), ranging from not detected to 2.5% relative abundance. The family was also diverse, represented by 220 unique ASVs. *Legionellaceae* was correlated with CO_2_, chloride, turbidity, noncarbonate hardness, sulfate, TDS, EC, magnesium, hardness, and calcium by the Spearman rank sum (α = 0.05). Overall, *Mycobacteriaceae* was detected in 49% of the samples (0–0.8% relative abundance except for 30% in well KM11) and was represented by 14 ASVs. Surprisingly, none of the potentially pathogenic families correlated with nitrate, in contrast to a different study of multiple aquifers in China.^14^

### Environmental Implications

Limitations of the study include the use of 16S amplicon sequencing, limiting taxonomic resolution compared to metagenomic sequencing, and preventing insight into function without metatranscriptomics, metabolomics, or proteomics. This study was also a single time point of sampling, which may limit applicability to other seasons, or even across years.^9^ The random forest regression was applied to limited data (few samples) and should primarily inform hypotheses for further investigation, such as Figure 5, rather than be taken as definitive or causative associations. Furthermore, well construction could include scenarios where well screens cross redox zones, which could further limit our results and interpretations. Finally, suspended microbial communities do not reflect the majority of aquifer biomass and can bias the apparent community structure, which may explain the low total correlation between hydrochemistry and microbiome composition.^7,78^

Detection of high concentrations of nontuberculous Mycobacteria and other pathogens, as well as nitrate and manganese above drinking water maximum contaminant levels, highlights the variability in alluvial aquifer quality and microbial composition. With poor knowledge about private well maintenance and water quality, there is an urgent need for characterization of potential source waters for private drinking water wells and for options to deliver safe drinking water, especially for vulnerable aquifers.

## Supplementary Material



## Notes

Additional table and supporting figures, including hyperparameters for grid search, additional taxonomy plots (Figure S6), an NMDS ordination of the Bray–Curtis distance among microbiomes by well-type plot (Figure S7), and an additional hydrochemical plot (Figure S8) (PDF)
