# Supplementary material for: Hydraulic Connectivity and Hydrochemistry Influence Microbial Community Structure in Agriculturally Affected Alluvial Aquifers in the Midwestern United States
Source: Environ Sci Technol. 2025 Jun 12;59(24):12279–91. doi: 10.1021/acs.est.5c03155 (PMC12199460; doi:10.1021/acs.est.5c03155)
Supplement: Supplementary file 1 [file es5c03155_si_001.pdf]

Supporting Information

Hydraulic connectivity and hydrochemistry influence microbial community structure in agriculturally affected alluvial aquifers in the Midwestern United States

*Hunter W. Schroer**,†,‡, *Kendra Markland*§, *Fangqiong Ling*Π, *and Craig L. Just*‡,⊥

†Civil, Architectural and Environmental Engineering, Missouri University of Science and Technology, Rolla, MO, 65409, USA

‡IIHR – Hydroscience and Engineering, University of Iowa, Iowa City, IA, 52240, USA

§United States Geological Survey, Iowa City, IA, 52240, USA

ΠDepartment of Energy, Environmental, & Chemical Engineering, Washington University in St. Louis, St. Louis, MO, 63130, USA

⊥Department of Civil & Environmental Engineering, University of Iowa, Iowa City, IA, 52240, USA

Summary: 7 pages, 1 table, 8 figures

Table of Contents

Table S1 .... S2
Figure S1 .... S2
Figure S2 .... S3
Figure S3 .... S4
Figure S4 .... S5
Figure S5 .... S5
Figure S6 .... S6
Figure S7 .... S6
Figure S8 .... S7

Table S1. Random forest grid search hyperparameters.

Parameter	Values searched	Optimal values
n_estimators	250, 500	250
max_depth	None, 2,3,4	None
min_samples	2,3,4	2
ccp_alpha	0,1,10,20	0

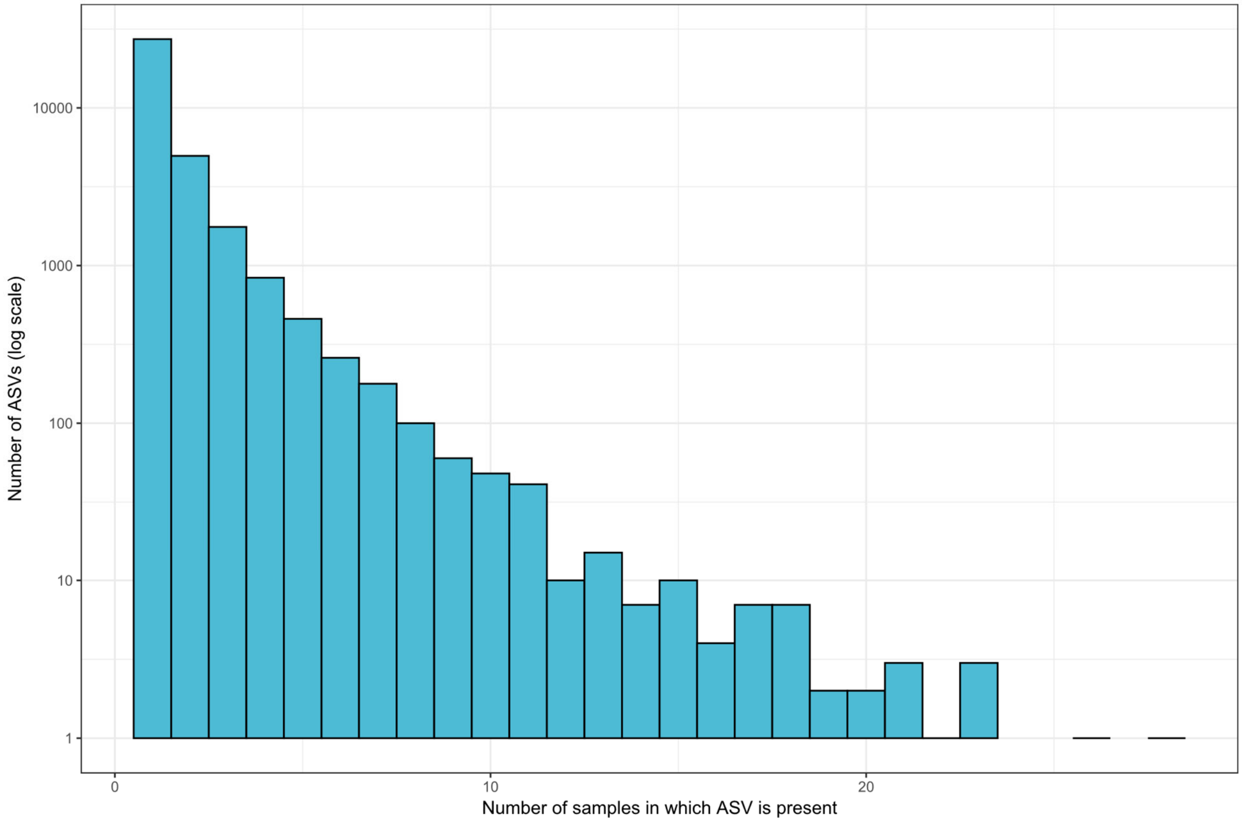

Figure S1. Frequency distribution of amplicon sequence variants (ASVs) across the groundwater samples.

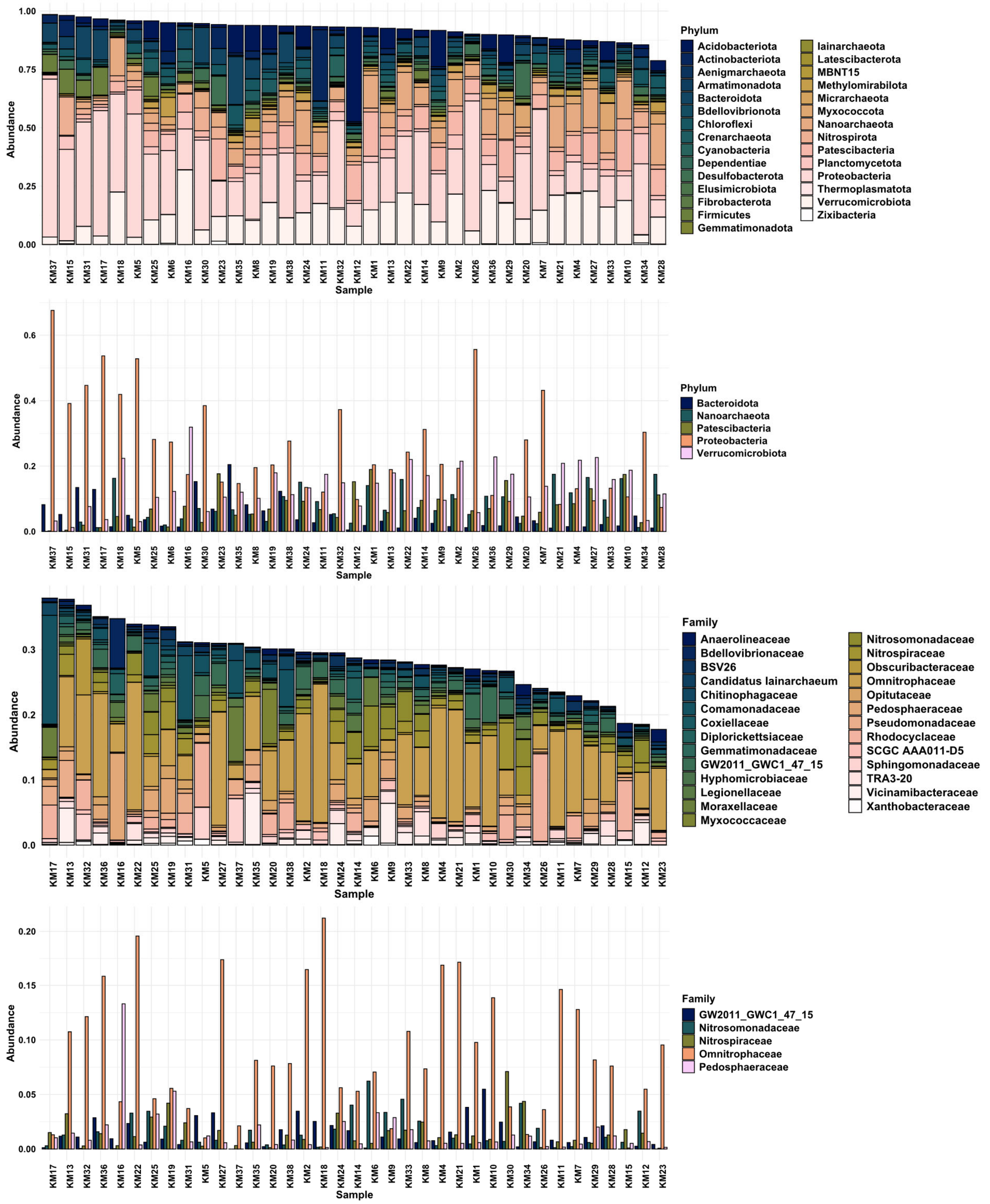

Figure S2. Microbiomes across the samples. A) Phyla present in more than 80% of samples. B) Five phyla with the highest median relative abundance across all samples. C) Families present in more than 90% of samples. B) Five families with the highest median relative abundance across all samples.

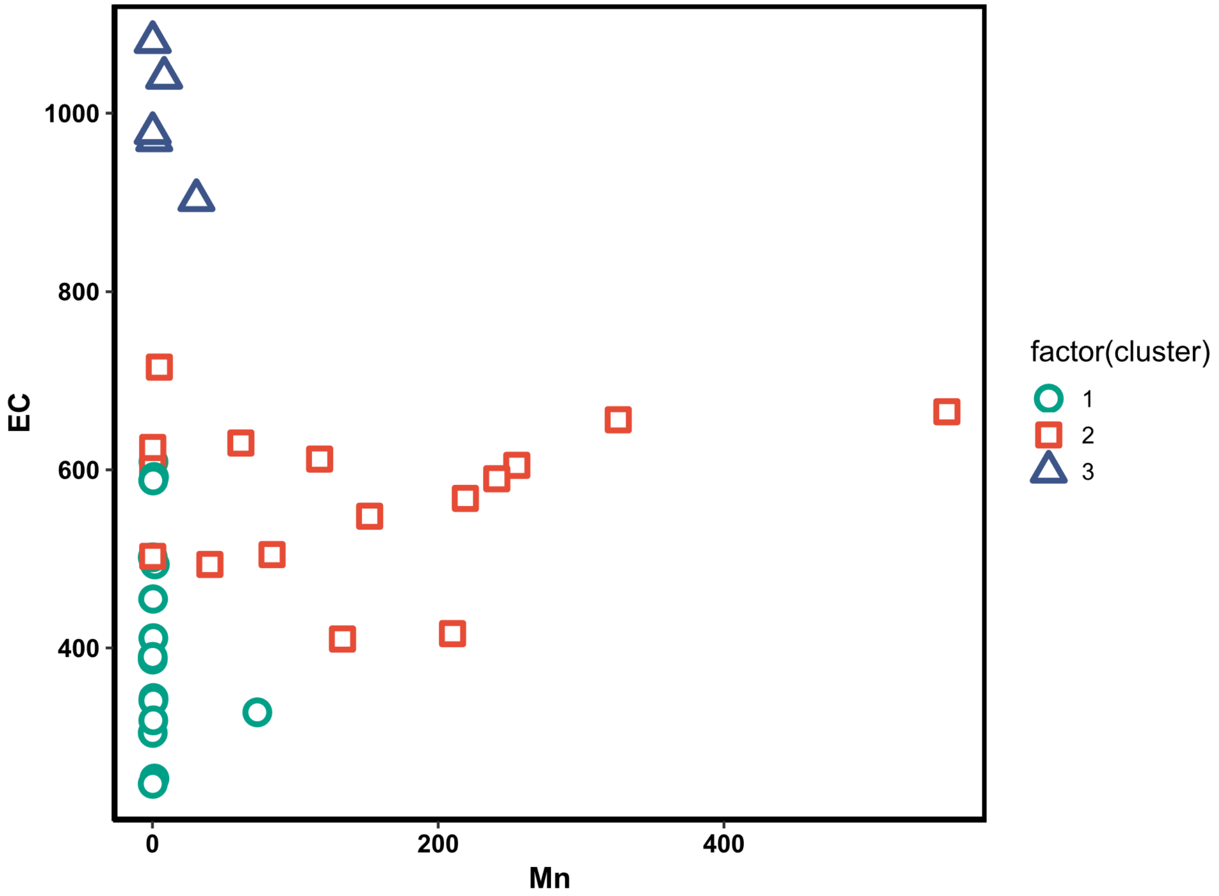

Figure S3. Measured values of specific conductance (EC) vs. dissolved manganese (Mn, in micrograms per liter). Clusters were determined from agglomerative hierarchical feature clustering (Ward's linkage) of the normalized hydrochemical dataset and are indicated by shape and color of the symbols, with each symbol representing a well sample. Unit for specific conductance is µS cm^{-1} – microSiemens per centimeter.

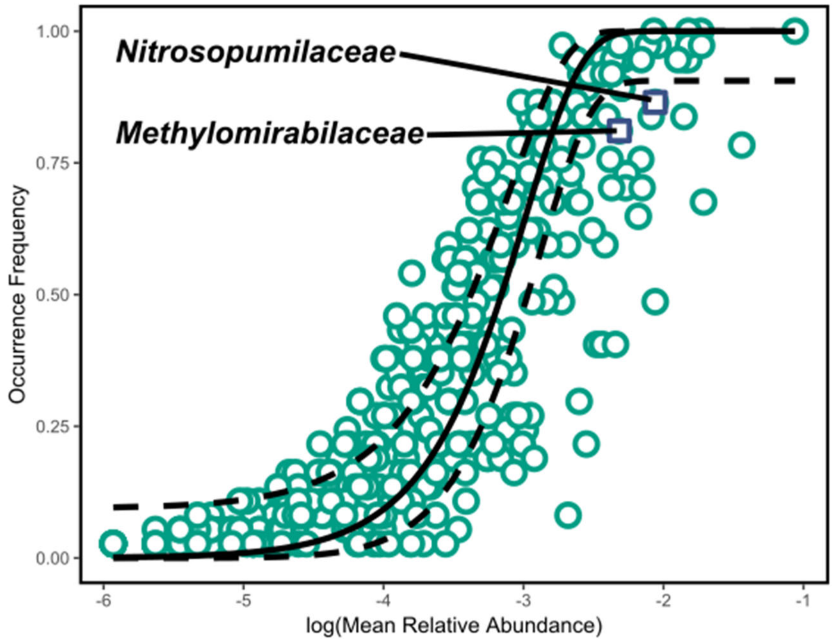

Figure S4. Neutral community modeling of family occurrence (prevalence) as a function of mean relative abundance. Each symbol is a single family across all 37 samples. Solid line is the beta distribution fit and the dashed lines are the 95% confidence interval.

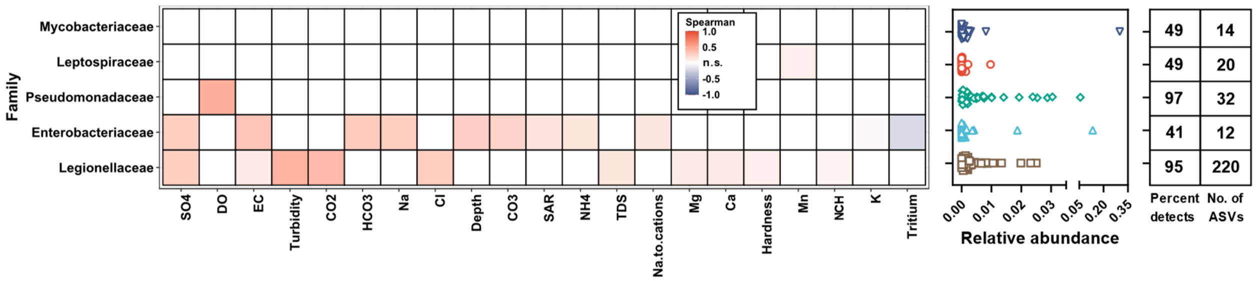

Figure S5. Summary of data on five families related to opportunistic pathogens, including Spearman rank sum correlation heatmap between pathogenic families and hydrochemical parameters, relative abundance of families across the samples, and frequency of detection of each family and number of ASVs assigned to each family. SO4 – sulfate, DO – dissolved oxygen, EC – specific conductance, CO2 – carbon dioxide, HCO3 – bicarbonate, Na – sodium, Cl – chloride, CO3 – carbonate, SAR – sodium adsorption ratio, NH4 – ammonia (ammonia + ammonium), TDS – total dissolved solids, Na.to.cations – sodium ratio of total cations, Mg – magnesium, Ca – calcium, Mn – manganese, NCH – non-carbonate hardness, K – potassium, ASV – amplicon sequencing variant.

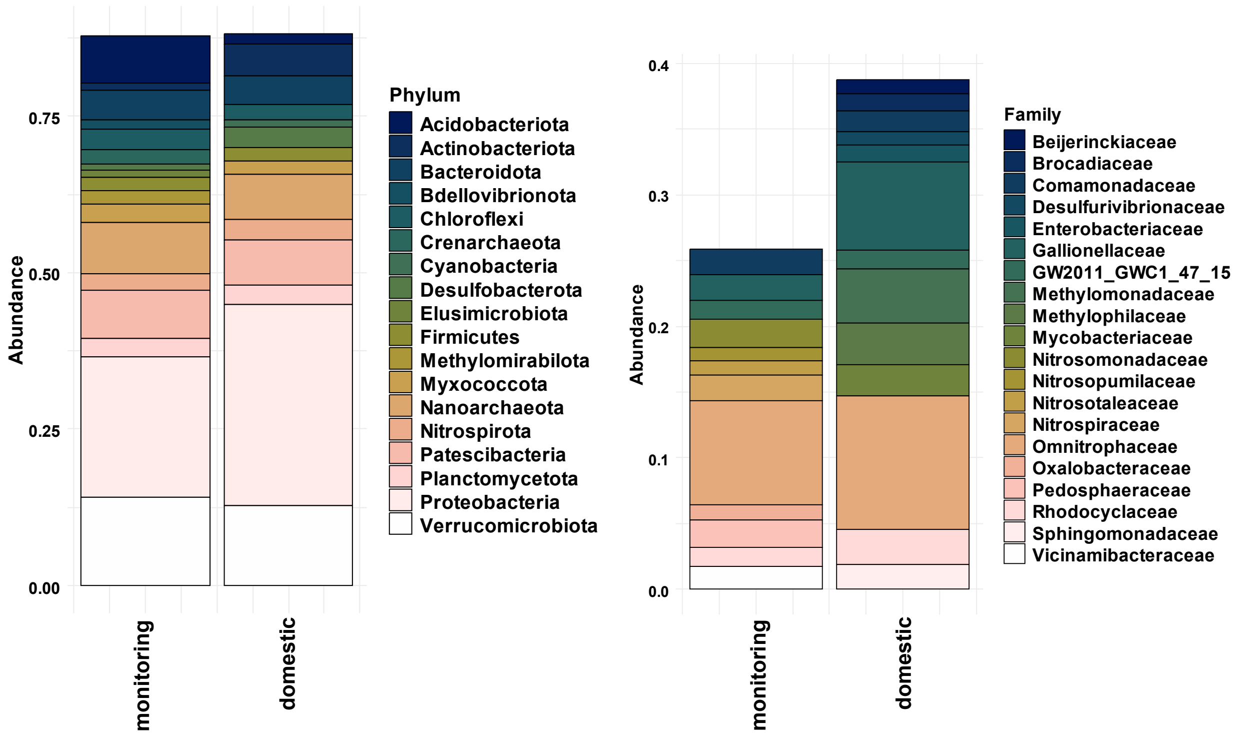

Figure S6. Mean relative abundance of each phylum (left) and family (right) grouped by monitoring or domestic wells.

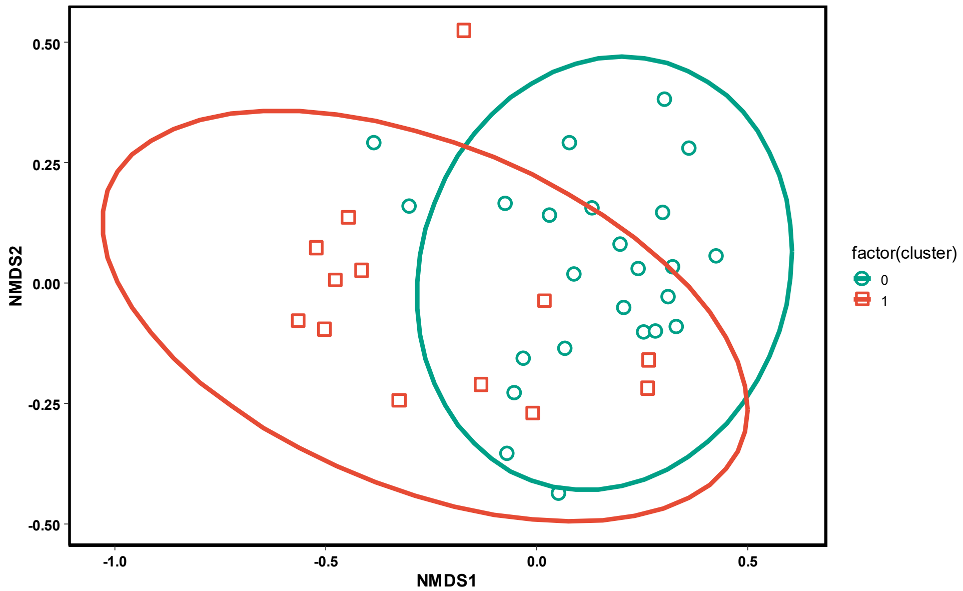

Figure S7. Non-metric multi-dimensional scaling (NMDS) ordination of Bray-Curtis distance among microbiomes. Symbols and colors represent monitoring (0, green circles) or drinking water wells (1, red squares), with each symbol representing a single well sample. Ellipses are 95% confidence ellipse of the respective cluster.

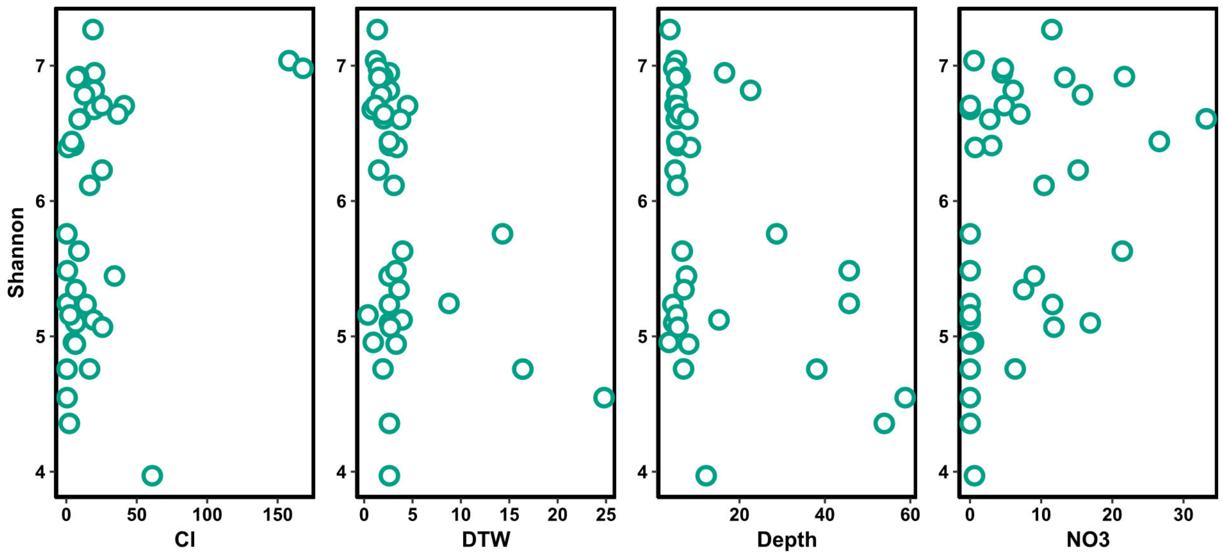

Figure S8. Correlation of hydrochemical variables with Shannon index (Spearman, α = 0.05): chloride (ρ = 0.42, p = 0.009), depth to water (ρ = -0.40, p = 0.014), well depth (ρ = -0.39, p = 0.018), and nitrate (ρ = 0.34, p = 0.042). Cl - chloride, DTW - depth to water, NO3 - nitrate.
